# Supplementary material for: Highly selective and robust single-atom catalyst Ru1/NC for reductive amination of aldehydes/ketones
Source: Nat Commun. 2021 Jun 2;12:3295. doi: 10.1038/s41467-021-23429-w (PMC8172939; doi:10.1038/s41467-021-23429-w)
Supplement: Supplementary file 1 — Supplementary Information [file 41467_2021_23429_MOESM1_ESM.pdf]

Highly Selective and Robust Single-Atom Catalyst Ru_1/NC for Reductive Amination of Aldehydes/Ketones

Haifeng Qi, et al.

Supplementary Methods

Catalyst preparation. Typically, a mixture of dicyandiamide ($C_2H_4N_4$) (12 g), L-cysteine ($C_3H_7NO_2S$) (3 g) and Ru(III) acetylacetonate ($Ru(acac)_3$) (40 mg) were added to a planetary ball mill pot, and were milled at a speed of 400 rpm for 2.0 h for twice at room temperature. The obtained fine powder was then subjected to temperature programmed pyrolysis in a tubular furnace under N_2 atmosphere with a flow rate of 60 ml min^{-1} and a two-stage heating program: the first stage began from 25 to 600 ℃ at a ramping rate of 3 ℃ min^{-1} and maintained at 600 ℃ for 2 h, and the second stage began from 600 to 900 ℃ at a ramping rate of 2 ℃ min^{-1} and maintained at 900 ℃ for 1 h. The as-made sample is denoted as Ru_1/NC-900. Other Ru_1/NC samples were synthesized by the same procedure except for different maximum temperatures. Ru_1/NC-900-800NH_3 was prepared by subjecting the Ru_1/NC-900 to a second pyrolysis treatment at 800 ^oC for 30 min with a ramping rate of 5 ℃ min^{-1} under NH_3/He mixture atmosphere (30 ml min^{-1} He and 20 ml min^{-1} NH_3).

$Ru_1/NC/Nb_2O_5$ catalyst was synthesized by the same procedure as Ru_1/NC-900-800NH_3, except for the addition of 2.00 g Nb_2O_5 (home-made) to the initial precursor mixture.

Ru/AC catalyst was prepared by a wet impregnation method. 1g of activated carbon was added into an appropriate amount of $RuCl_3$ solution (0.32 mg mL^{-1} Ru) in continuous stirring at room temperature for 10 min. Then the suspension was dried overnight at 80 ^oC. The obtained sample was ground into powder and reduced at 300 ℃ for 2 h by pure H_2 with a flow rate of 60 mL min^{-1}.

1wt% Ru/Nb_2O_5 catalyst was prepared by impregnation method according to the reported procedure.[1] 2.0 g of home-made Nb_2O_5 was dispersed into 50 mL of 4.0 mM $Ru(NO)(NO_3)_3$ solution under stirring for 12 h. Then the suspension was slowly evaporated at 60 ^oC via rotary evaporator until drying, followed by vacuum drying at 100 ^oC for 1 h. The obtained sample was ground to powder and reduced under 5% H_2/Ar flow (60 mL/min) at 400 ^oC for 2 h. After being cooled to room temperature,

the catalyst was passivated with 1% (v/v) O_2/N_2 for 8 h.

5wt% Ru/HZSM-5 catalyst was prepared according to the reported procedure.[5] 1.0 g HZSM-5 (molar ratio of SiO_2/Al_2O_3 = 46) was mixed with 1.50 g solution of $RuCl_3$ (0.32 mg mL^{-1} Ru). Then the mixture was slowly evaporated at 60 °C under stirring and dried at 80 ℃ for 12 h. The obtained sample was calcined at 400 ℃ for 4 h and reduced at 300 ℃ for 2 h under H_2 atmosphere. After being cooled to room temperature, the catalyst was passivated with 1% (v/v) O_2/N_2 for 8 h.

Catalytic tests. The reductive amination reaction was conducted in a 50 mL stainless-steel autoclave (Parr Instrument Company, America). Typically, the substrate (2 mmol), Ru_1/NC catalyst (molar ratio of Ru: substrate was 1:400), dodecane (internal standard) and 3 mL methanol (solvent) were added to a teflon lining. Then the autoclave was sealed and purged by N_2 for three times, followed by charging with 0.5 MPa NH_3 and 2 MPa H_2. The reaction mixture was stirred at 100 ℃ for 10 h. The products were identified using a Varian G450/320 GC/MS system, and were quantitatively analyzed using an Agilent 7890A GC system equipped with a HP-5 capillary column and a FID detector.

Purification experiment. Selected primary amine products were purified by column chromatography (silica; ethyl acetate- methanol mixture). The purified amines were converted to their corresponding hydrochloride salt by reacting with 2.0 M HCl followed by water removal through rotary evaporator for characterization by NMR and FT-ICR-MS spectral analysis.

Characterization methods.

In the sample characterization section, the actual Ru loadings were determined by inductively coupled plasma spectroscopy (ICP-OES) on an IRIS Intrepid II XSP instrument (Thermo Electron Corporation).

X-ray diffraction (XRD) patterns were recorded on a PANalytical X' pert diffractometer with a Cu-Kα radiation source (40 kV and 40 mA). A continuous mode was used to record data in the 2θ range from 10° to 80°.

N_2 adsorption-desorption experiments were conducted on a Micromeritics ASAP-2010 physical adsorption apparatus. Before the measurement, the sample was

pretreated at 110 ^{o}C for 2 h and 300 ^{o}C for 4 h in vacuum. The specific surface area was calculated using a Brunauer-Emmett-Teller (BET) method.

Scanning transmission electron microscopy (STEM) and energy dispersive X-ray spectroscopy (EDS) experiments were performed on a JEOL JEM-2100F microscope operated at 200 kV, equipped with an Oxford Instruments ISIS/INCA energy-dispersive X-ray spectroscopy (EDS) system with an Oxford Pentafet Ultrathin Window (UTW) Detector. The aberration-corrected high-angle annual dark-filed scanning transmission electron microscopy (AC-HAADF-STEM) analysis was performed on a JEOL JEM-ARM200F equipped with a CEOS probe corrector, with a guaranteed resolution of 0.08 nm. The electron energy loss spectroscopy (EELS) analysis of Ru_1/NC-800-900NH_3 was performed on a Field Emission HF5000 Microscope (200 kV accelerating voltage; aberration corrector, 0.078 nm spatial resolution). Before microscopy examination, the sample was ultrasonically dispersed in ethanol for 15-20 min, and then a drop of the suspension was dropped on a copper TEM grid coated with a thin holey carbon film.

X-ray photoelectron spectroscopy (XPS) spectra were obtained on a Thermo ESCALAB 250 X-ray photoelectron spectrometer equipped with Al Kα excitation source and with C as internal standard (C 1s = 284.6 eV).

The X-ray absorption spectra (XAS) including X-ray absorption near edge structure (XANES) and extended X-ray absorption fine structure (EXAFS) at Ru K-edge of the samples were measured at the beamline 14W of Shanghai Synchrotron Radiation Facility (SSRF) in China. The output beam was selected by Si(311) monochromator, and the energy was calibrated by Ru foil. The data were collected at room temperature under transmission mode. Athena software package was employed to process the XAS data.

Microcalorimetric measurement was performed by a BT2.15 heat-flux calorimeter, which was connected to a gas handling and a volumetric system employing MKS Baratron Capacitance Manometers for precision pressure measurement. The ultimate dynamic vacuum of the microcalorimetric system was 10^{-7} Torr by calculation. First, the fresh sample was treated in a special tube in H_2 at

100 ^{o}C for 1h and then high pure He at 200 ^{o}C for 1h to eliminate the adsorption. Then, the tube was transferred into the the high vacuum system and stabilized for (6-8 h). After thermal equilibrium was reached, the H_2-microcalorimetric data was collected by sequentially introducing small doses (10^{-6} mol) of H_2 (CO_2 or NH_3) into the system until it became saturated (5-6 Torr). Simultaneously, the differential heat versus adsorbate coverage plots and adsorption isothermals can be obtained after a typical microcalorimetric experiment.

NMR spectra of selected products were recorded at room temperature in C_2D_6OS on 400 MHz Bruker DRX-400 NMR and 700 MHz Bruker DRX-700 NMR (Table 3, Entry 13) spectrometers. Fourier transform-ion cyclotron resonance-mass spectra (FT-ICR-MS) of selected products were obtained on SolariX XR-15T FTMS of Bruker.

Supplementary Figure 1

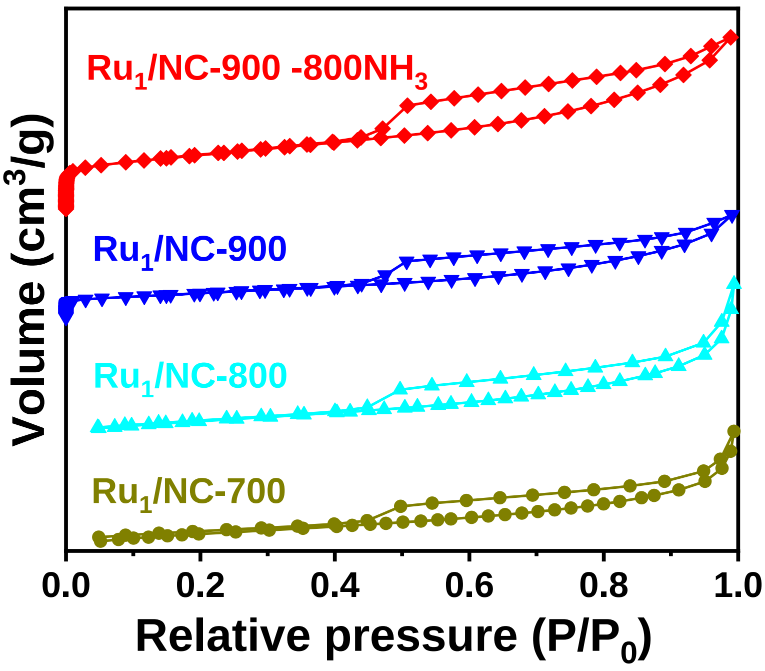

Supplementary Figure 1. The adsorption-desorption isotherms of Ru_1/NC-T samples

Supplementary Figure 2

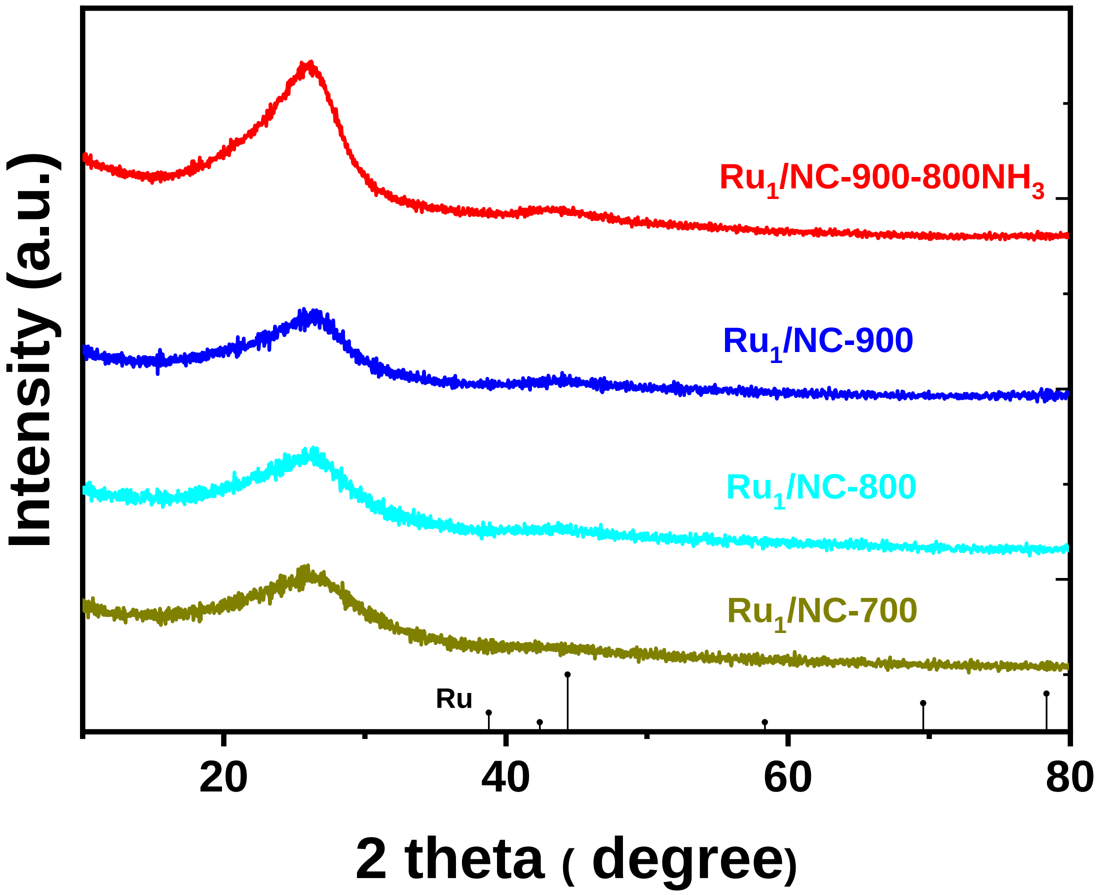

Supplementary Figure 2. XRD patterns of Ru_1/NC-T samples

Supplementary Figure 3

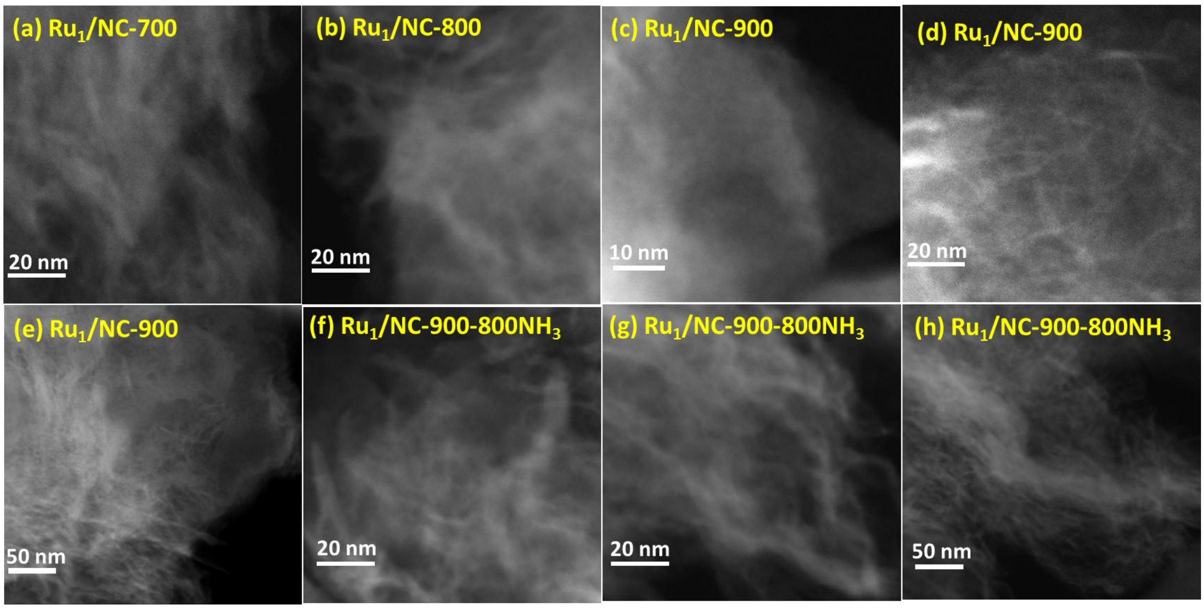

Supplementary Figure 3. Low-magnification STEM images of Ru_1/NC-T samples. **a** Ru_1/NC-700. **b** Ru_1/NC-800. **c-e** Ru_1/NC-900. **f-h** Ru_1/NC-900-800NH_3.

Supplementary Figure 4

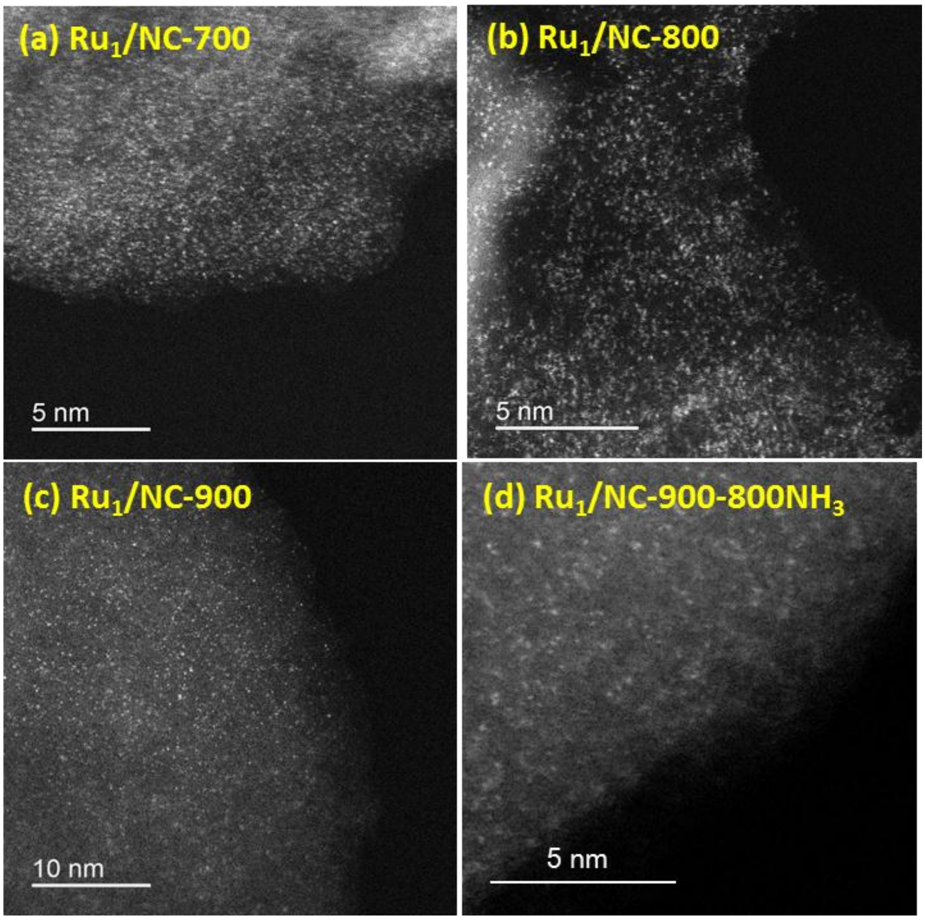

Supplementary Figure 4. Aberration-corrected HAADF-STEM images of Ru_1/NC-T samples. **a** Ru_1/NC-700. **b** Ru_1/NC-800. **c** Ru_1/NC-900. **d** Ru_1/NC-900-800NH_3.

Supplementary Figure 5

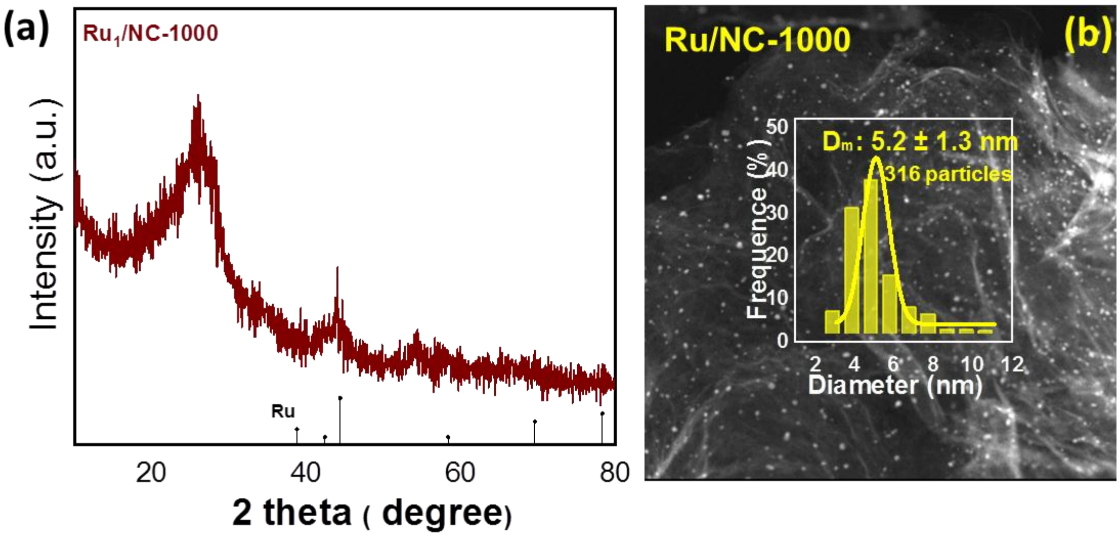

Supplementary Figure 5. Characterizations of Ru/NC-1000 sample. **a** XRD pattern. **b** HAADF-STEM image.

Supplementary Figure 6

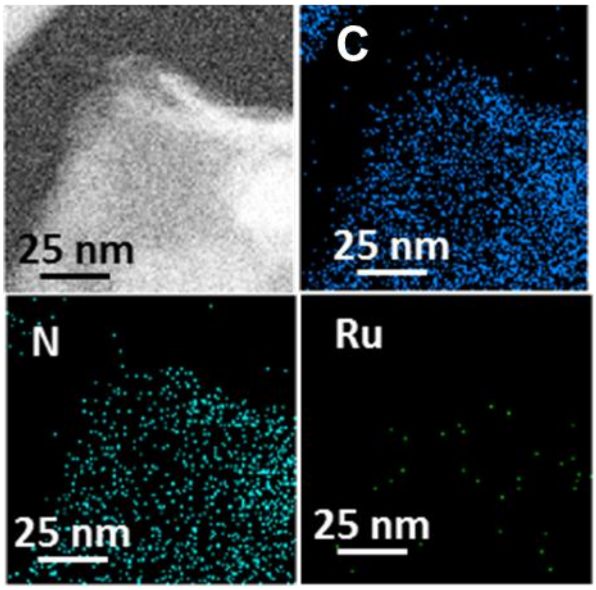

Supplementary Figure 6. EDS mapping of Ru_1/NC-900-800NH_3 sample

Supplementary Figure 7

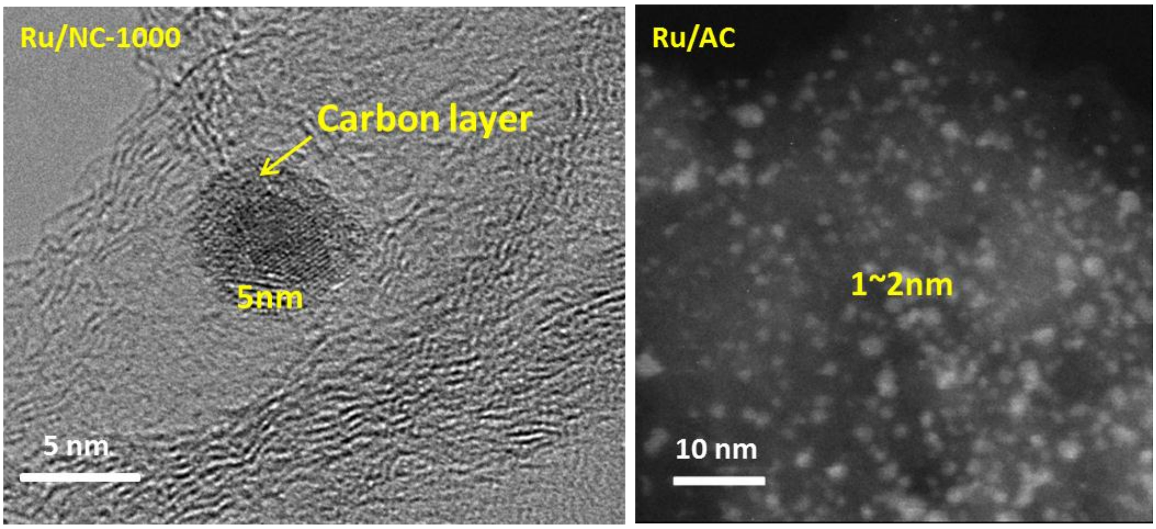

Supplementary Figure 7. HRTEM images of Ru/NC-1000 and Ru/AC samples

Supplementary Figure 8

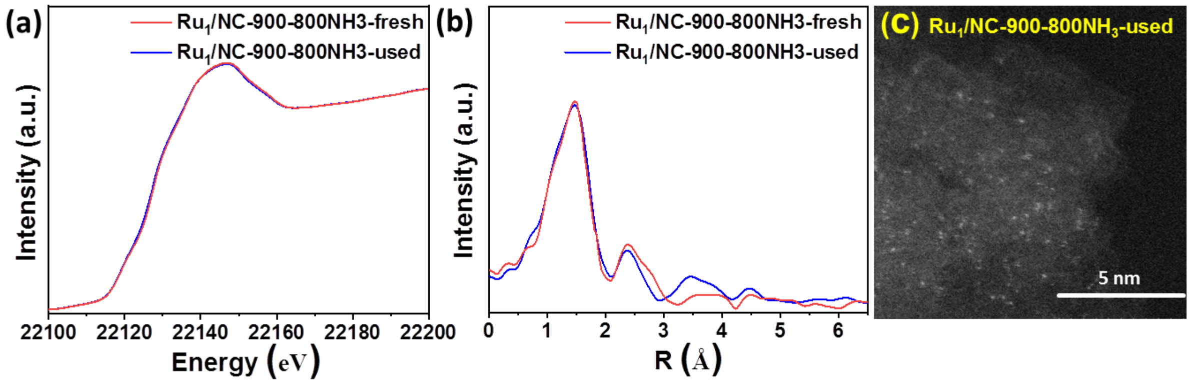

Supplementary Figure 8. Characterizations of fresh and used Ru_1/NC-900-800NH_3. **a** The normalized X-ray absorption near-edge spectra at the Ru K-edge. **b** the k^2-weighted Fourier transform spectra. **c** High-angle annular dark-field scanning transmission electron microscopy image of the used Ru_1/NC-900-800NH_3.

Supplementary Figure 9

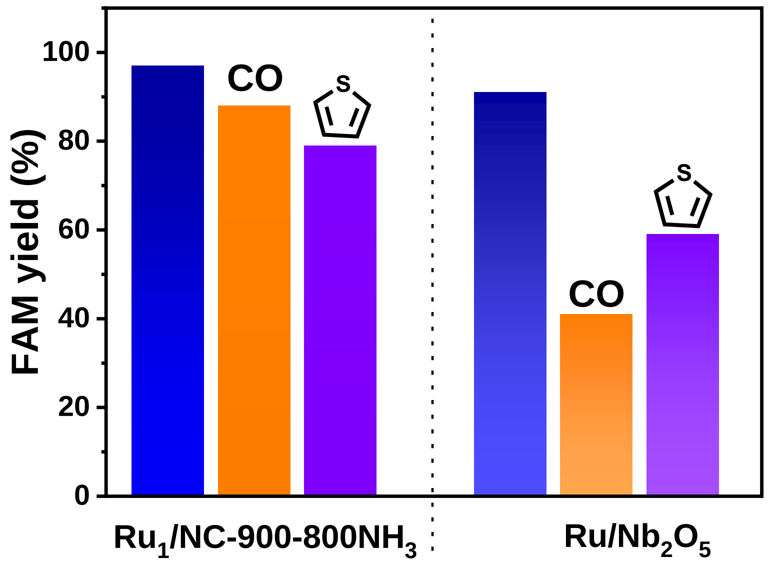

Supplementary Figure 9. Poisoning experiments of Ru_1/NC-900-800NH_3 and Ru/Nb_2O_5 by CO and sulfur. Reaction condition: 2 mmol furfural, 22 mg Ru_1/NC-900-800NH_3 or 51 mg Ru/Nb_2O_5 to maintain the molar ratio of Ru: furfural = 1: 400, 3 g methanol, 0.5 MPa NH_3, 2 MPa H_2, 100 ℃, 10 h. For CO poisoning experiment, 2 MPa 1vol% CO in H_2 was used in place of pure H_2; for sulfur poisoning experiment, 500 ppm thiophene was added to the reaction mixture.

Supplementary Figure 10

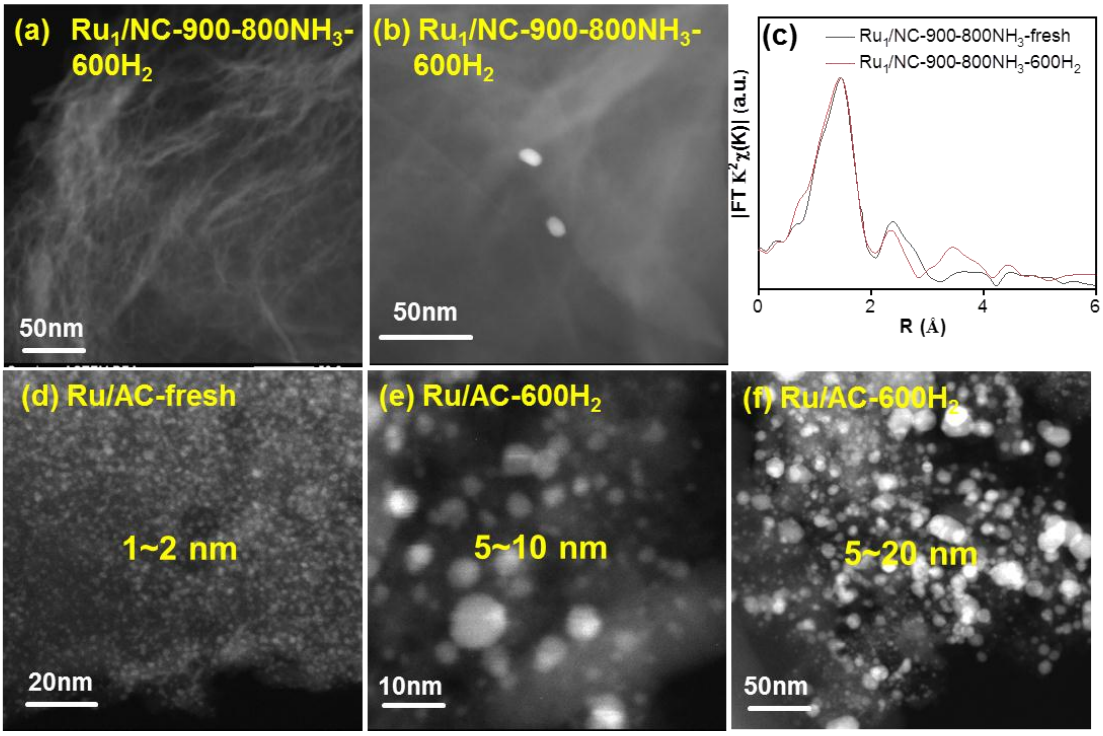

Supplementary Figure 10. Characterizations of Ru_1/NC-900-800NH_3 and Ru/AC. **a, b** HAADF-STEM images of Ru_1/NC-900-800NH_3 after hydrogen treatment at 600 °C. **c** the k^2-weighted Fourier transform spectra at r-space of Ru_1/NC-900-800NH_3 before and after hydrogen treatment at 600 °C. **d-f** High-angle annular dark-field scanning transmission electron microscopy images of Ru/AC before and after hydrogen treatment at 600 °C.

Supplementary Figure 11

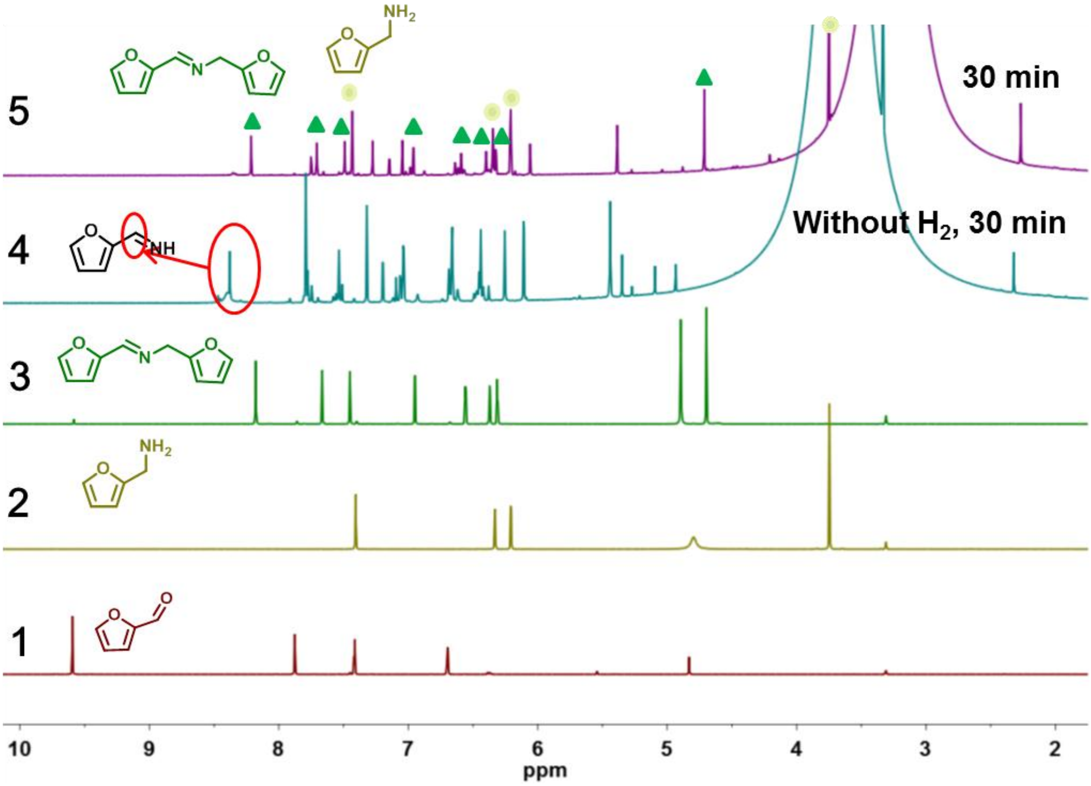

Supplementary Figure 11. In-situ ^{1}HNMR during the reductive amination of furfural. 1. Furfural; **2**. Furfuryl amine; **3**. Schiff base. **4**. 2 mmol furfural, 22 mg Ru_1/NC-900-800NH_3 (0.25mol% Ru, molar ratio of Ru : furfural = 1: 400), 0.5 MPa NH_3, 100 ℃, 30 min, dodecane used as internal standard; **5**. 2 mmol furfural, 22 mg Ru_1/NC-900-800NH_3 (0.25mol% Ru, molar ratio: Ru : furfural=1: 400), 0.5 MPa NH_3, 2 MPa H_2, 100 ℃, 30 min, dodecane used as internal standard.

Supplementary Figure 12

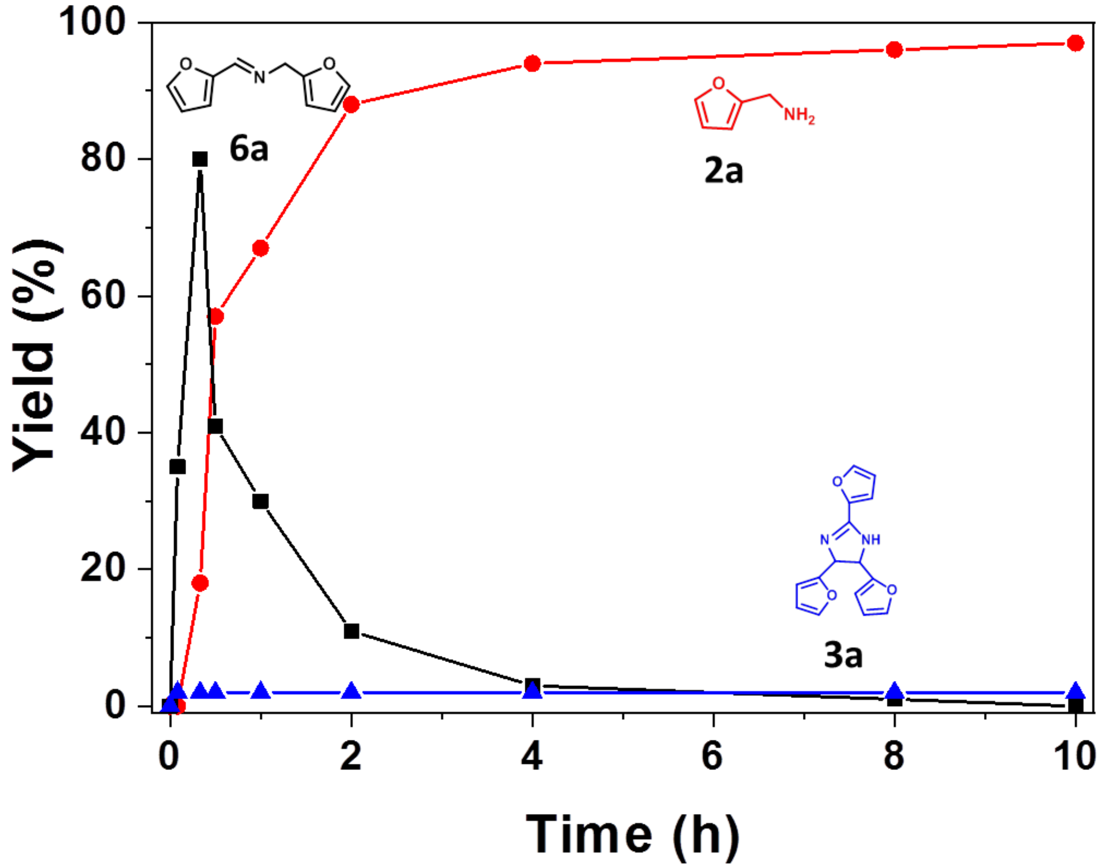

Supplementary Figure 12. Kinetic profile for reductive amination of furfural over Ru_1/NC-900-800NH_3 catalyst. Reaction condition: 2 mmol furfural, 22 mg Ru_1/NC-900-800NH_3 (0.25mol% Ru, molar ratio of Ru : furfural = 1 : 400), 0.5 MPa NH_3, 2 MPa H_2, 100 ℃, dodecane as the internal standard.

Supplementary Figure 13

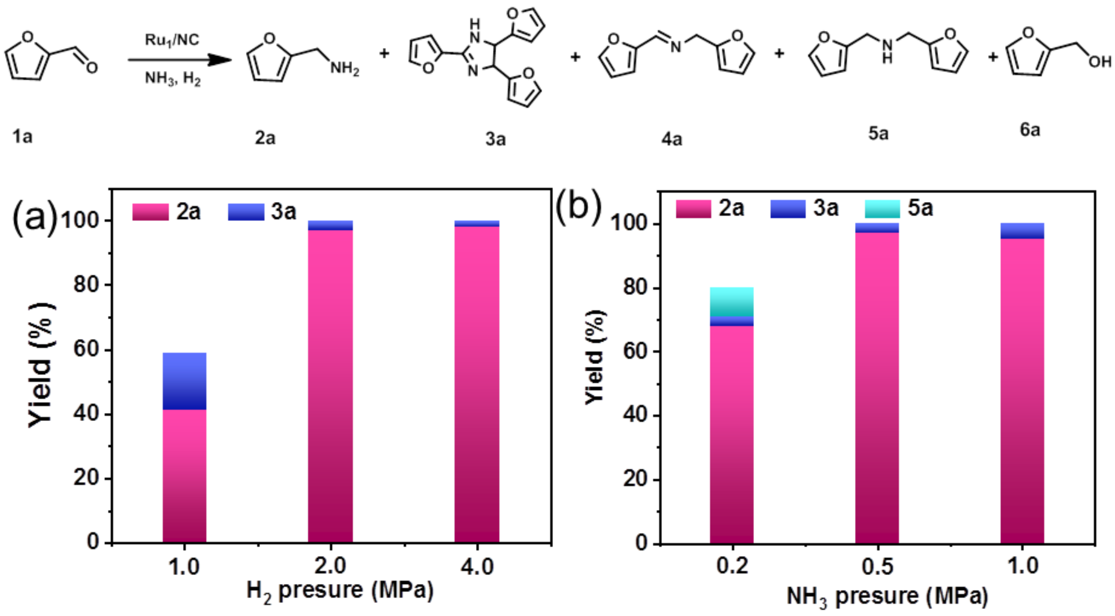

Supplementary Figure 13. Effects of reaction parameters on the FAM yield in the reductive amination of furfural. **a** H_2 pressure. **b** NH_3 pressure. Reaction condition: 2 mmol furfural, 22 mg Ru_1/NC-900-800NH_3 catalyst (0.25mol% Ru, molar ratio of Ru: furfural = 1: 400), 3 g methanol, 100 ℃, 10 h, dodecane as the internal standard.

Supplementary Figure 14

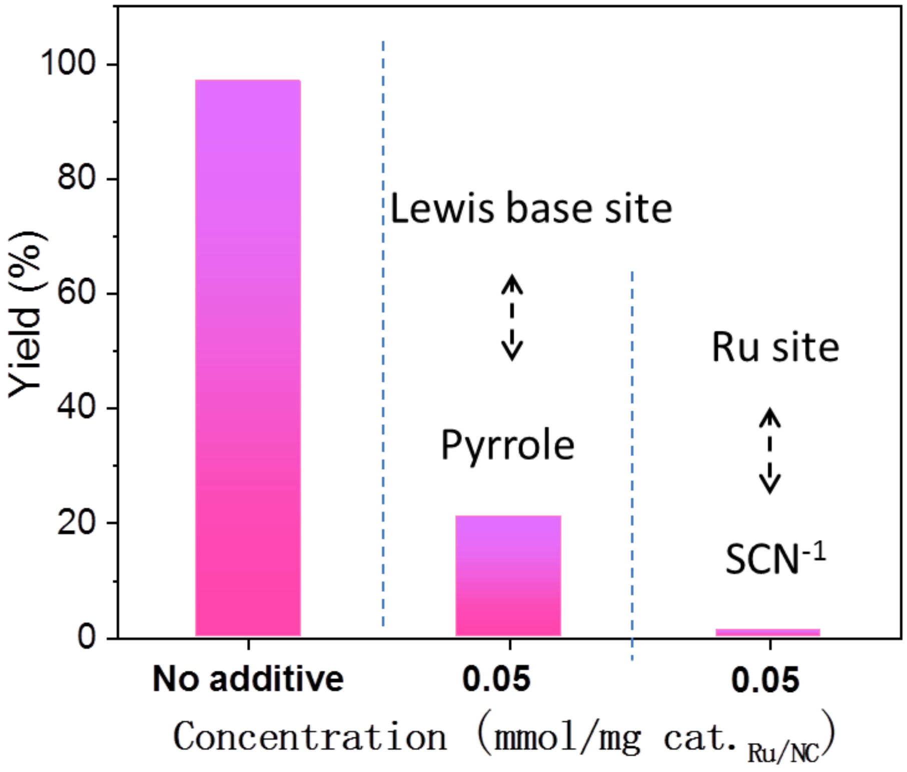

Supplementary Figure 14. The effect of Lewis acid additive (pyrrole) and Lewis base additive (KSCN) on the FAM yield in reductive amination of furfural. Before the reaction, 0.05 mmol/mg_{Cat} of KSCN or pyrrole was injected into the reaction system. Reaction conditions: 2 mmol furfural, 22 mg Ru_1/NC-900-800NH_3 (0.25mol% Ru, molar ratio of Ru : furfural = 1 : 400), 3 g methanol, 0.5 MPa NH_3, 2 MPa H_2, 100 ℃, 10h, dodecane as the internal standard.

Supplementary Figure 15

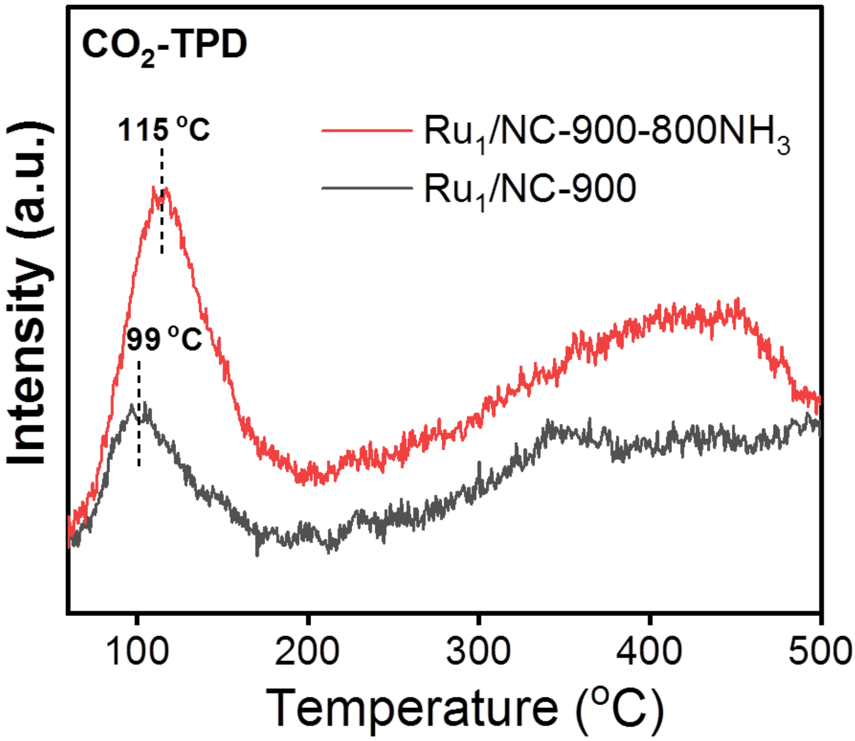

Supplementary Figure 15. CO_2-TPD profile of Ru_1/NC-900 and Ru_1/NC-900-800NH_3 catalysts.

Supplementary Figure 16

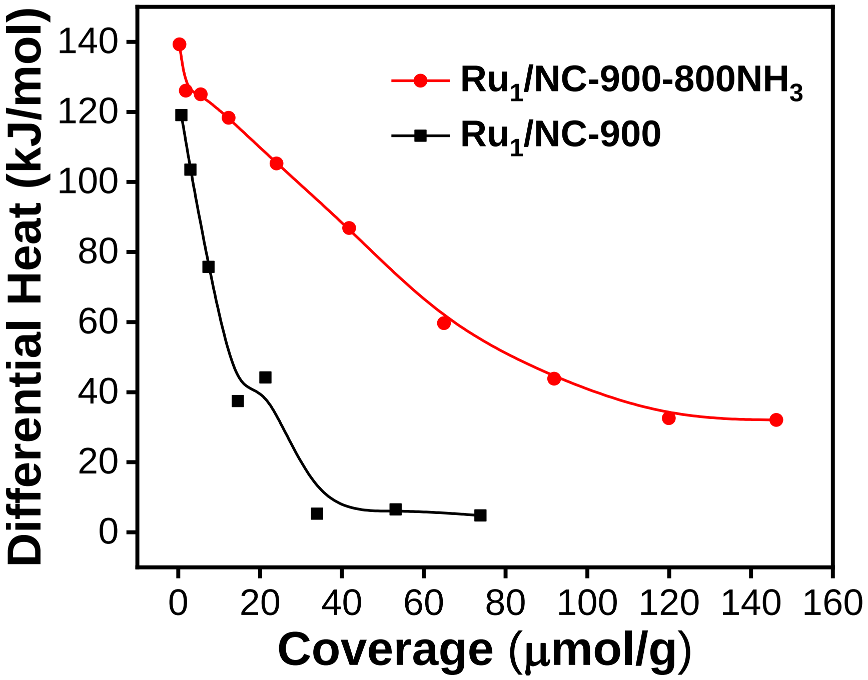

Supplementary Figure 16. Differential adsorption heat of CO_2 on Ru_1/NC-900 and Ru_1/NC-900-800NH_3 catalysts as a function of coverage.

Supplementary Figure 17

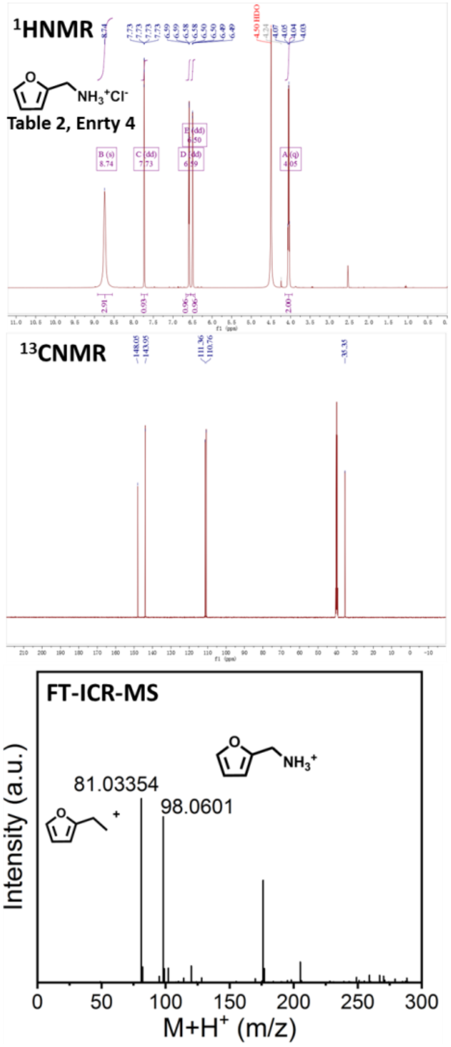

^{1}H NMR (400 MHz, DMSO-d_6) δ 8.74 (s, 3H), 7.73 (dd, J = 1.9, 0.8 Hz, 1H), 6.59 (dd, J = 3.3, 0.8 Hz, 1H), 6.50 (dd, J = 3.3, 1.8 Hz, 1H), 4.05 (q, J = 5.8 Hz, 2H). ^{13}C NMR (101 MHz, DMSO) δ 148.05, 143.95, 111.36, 110.76, 35.35. FT-ICR-MS (m/z): Calcd for $C_5H_8NO^+$ $[M+H]^+$ 98.0600; found 98.0601.

Supplementary Figure 17. ^{1}H NMR, ^{13}C NMR and FT-ICR-MS spectra of the primary amine product (Table 2, Entry 4).

Supplementary Figure 18

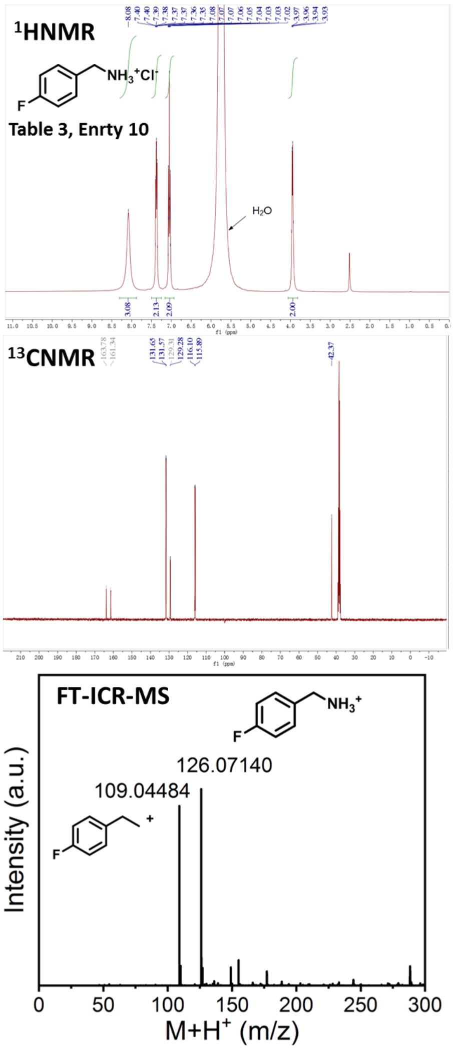

^{1}H NMR (400 MHz, DMSO-d_6) δ 8.08 (s, 3H), 7.38 (m, J = 9.0, 5.7, 2.9 Hz, 2H), 7.11 – 7.00 (m, 2H), 3.95 (q, J = 5.9 Hz, 2H). ^{13}C NMR (101 MHz, DMSO) δ 163.78, 161.34, 131.65, 131.57, 129.31, 129.28, 116.10, 115.89, 42.37. FT-ICR-MS (m/z): Calcd for $C_7H_9FN^+$ $[M+H]^+$ 126.07135; found 126.07140.

Supplementary Figure 18. ^{1}H NMR, ^{13}C NMR and FT-ICR-MS spectra of the primary amine product (Table 3, Entry 10).

Supplementary Figure 19

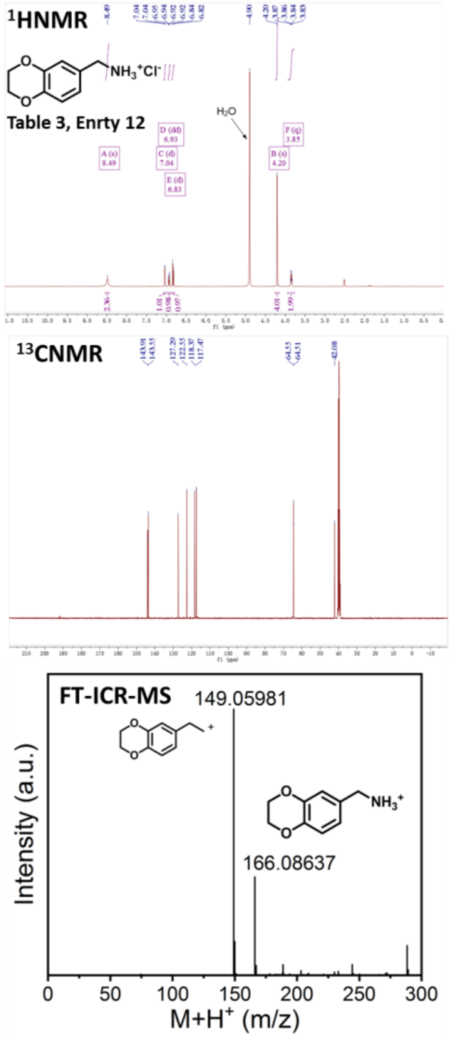

^{1}H NMR (400 MHz, DMSO-d_6) δ 8.49 (s, 3H), 7.04 (d, *J* = 2.1 Hz, 1H), 6.93 (dd, *J* = 8.3, 2.1 Hz, 1H), 6.83 (d, *J* = 8.3 Hz, 1H), 4.20 (s, 4H), 3.85 (q, *J* = 5.8 Hz, 2H). ^{13}C NMR (101 MHz, DMSO) δ 143.91, 143.55, 127.29, 122.55, 118.37, 117.47, 64.55, 64.51, 42.08. FT-ICR-MS (m/z): Calcd for $C_9H_{12}NO_2^+$ $[M+H]^+$ 166.08626; found 166.08637.

Supplementary Figure 19. ^{1}H NMR, ^{13}C NMR and FT-ICR-MS spectra of the primary amine product (Table 3, Entry 12).

Supplementary Figure 20

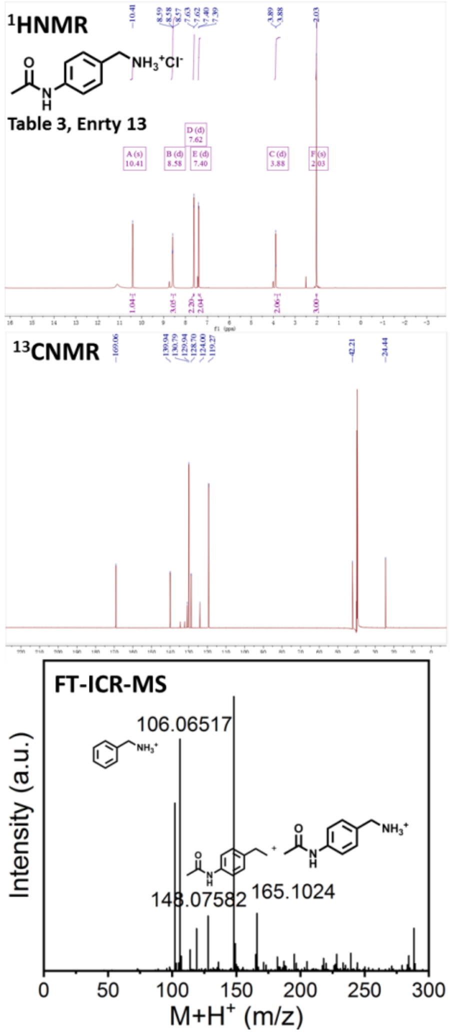

^{1}H NMR (700 MHz, DMSO-d_6) δ 10.41 (s, 1H), 8.58 (d, J = 6.5 Hz, 3H), 7.62 (d, J = 8.4 Hz, 2H), 7.40 (d, J = 8.4 Hz, 2H), 3.88 (d, J = 5.9 Hz, 2H), 2.03 (s, 3H). ^{13}C NMR (176 MHz, DMSO) δ 169.06, 139.94, 130.79, 129.94, 128.70, 124.00, 119.27, 42.21, 24.44. FT-ICR-MS (m/z): Calcd for $C_9H_{13}N_2O^+$ $[M+H]^+$ 165.10224; found 165.1024.

Supplementary Figure 20. ^{1}H NMR, ^{13}C NMR and FT-ICR-MS spectra of the primary amine product (Table 3, Entry 13).

Supplementary Figure 21

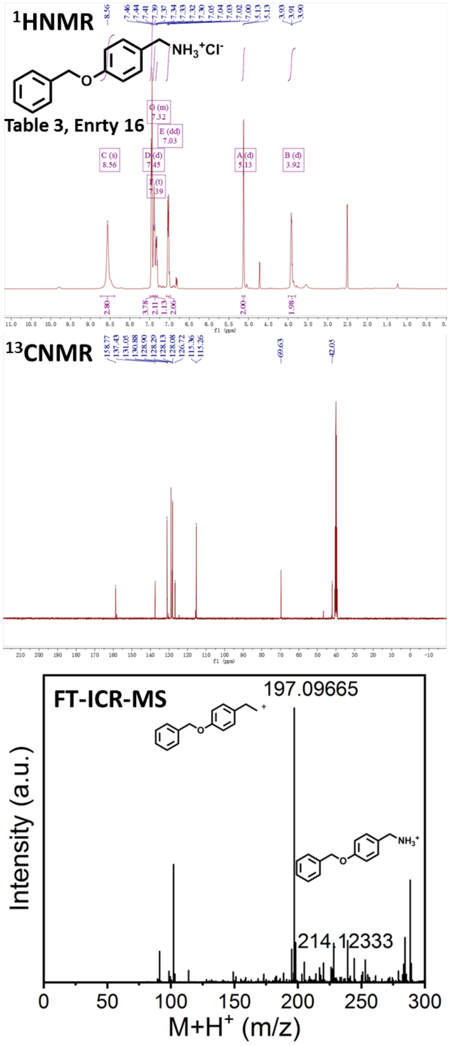

^{1}H NMR (400 MHz, DMSO-d_6) δ 8.56 (s, 3H), 7.45 (d, J = 8.4 Hz, 4H), 7.39 (t, J = 7.2 Hz, 2H), 7.35 – 7.30 (m, 1H), 7.03 (dd, J = 8.7, 2.8 Hz, 2H), 5.13 (d, J = 3.0 Hz, 2H), 3.92 (d, J = 5.8 Hz, 2H). ^{13}C NMR (101 MHz, DMSO) δ 158.77, 137.43, 131.05, 130.88, 128.90, 128.29, 128.13, 128.08, 126.72, 115.36, 115.26, 69.63, 42.05. FT-ICR-MS (m/z): Calcd for $C_{14}H_{16}NO^{+}$ $[M+H]^{+}$ 214.12264; found 214.12333.

Supplementary Figure 21. ^{1}H NMR, ^{13}C NMR and FT-ICR-MS spectra of the primary amine product (Table 3, Entry 16).

Supplementary Figure 22

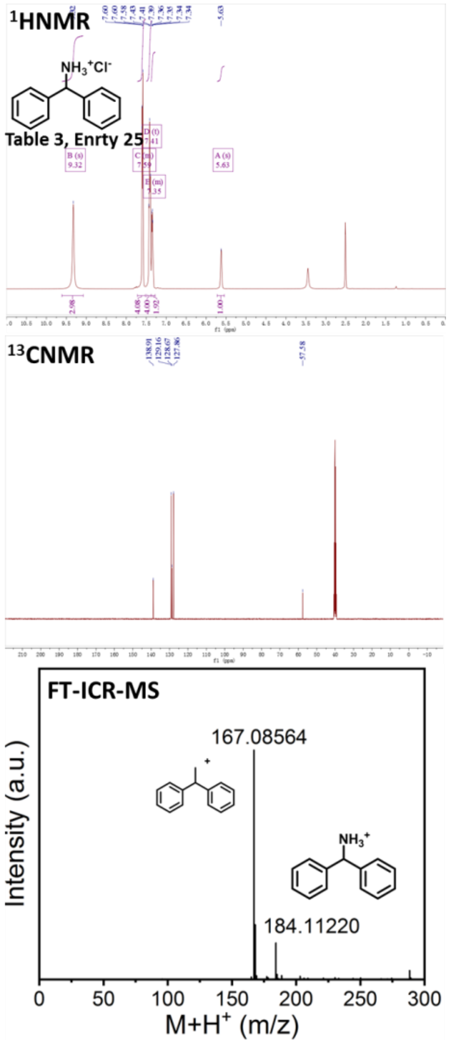

^{1}H NMR (400 MHz, DMSO-d_6) δ 9.32 (s, 3H), 7.72 – 7.52 (m, 4H), 7.41 (t, J = 7.5 Hz, 4H), 7.37 – 7.27 (m, 2H), 5.63 (s, 1H). ^{13}C NMR (101 MHz, DMSO) δ 138.91, 129.16, 128.67, 127.86, 57.58. FT-ICR-MS (m/z): Calcd for $C_{13}H_{14}N^+$ $[M+H]^+$ 184.11208; found 184.11220.

Supplementary Figure 22. ^{1}H NMR, ^{13}C NMR and FT-ICR-MS spectra of the primary amine product (Table 3, Entry 25).

Supplementary Figure 23

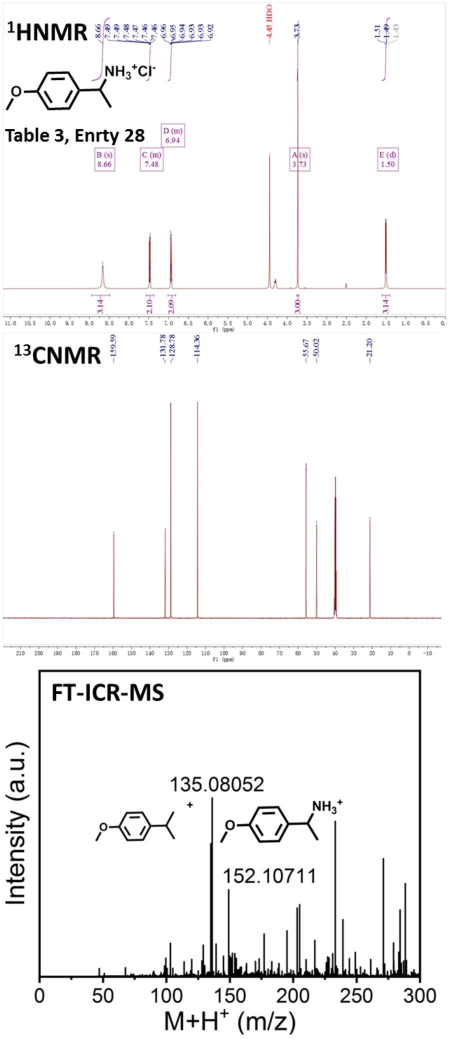

^{1}H NMR (400 MHz, DMSO-d_6) δ 8.66 (s, 3H), 7.57 – 7.37 (m, 2H), 7.02 – 6.82 (m, 2H), 3.73 (s, 3H), 1.50 (d, J = 6.8 Hz, 3H). ^{13}C NMR (101 MHz, DMSO) δ 159.59, 131.78, 128.78, 114.36, 55.67, 50.02, 21.20. FT-ICR-MS (m/z): Calcd for $C_9H_{14}NO^+$ $[M+H]^+$ 152.10699; found 152.10711.

Supplementary Figure 23. ^{1}H NMR, ^{13}C NMR and FT-ICR-MS spectra of the primary amine product (Table 3, Entry 28).

Supplementary Figure 24

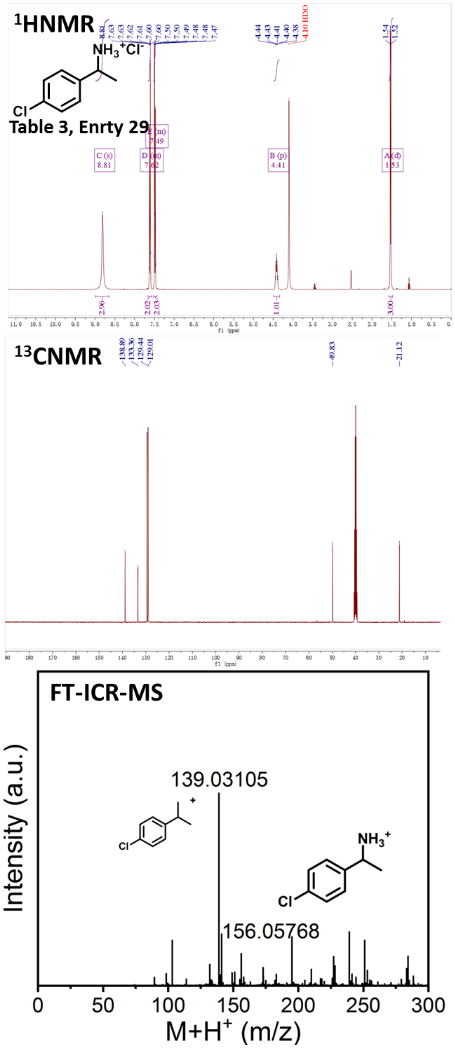

^{1}H NMR (400 MHz, DMSO-d_6) δ 8.81 (s, 3H), 7.66 – 7.57 (m, 2H), 7.53 – 7.44 (m, 2H), 4.41 (p, J = 6.1 Hz, 1H), 1.53 (d, J = 6.8 Hz, 3H). ^{13}C NMR (101 MHz, DMSO) δ 138.89, 133.36, 129.44, 129.01, 49.83, 21.12. FT-ICR-MS (m/z): Calcd for $C_8H_{11}ClN^+$ $[M+H]^+$ 156.05745; found 156.05768.

Supplementary Figure 24. ^{1}H NMR, ^{13}C NMR and FT-ICR-MS spectra of the primary amine product (Table 3, Entry 29).

Supplementary Table 1. The metal loading and the textural parameters of Ru_1/NC-T samples*

Catalysts	Ru Loading (wt%)	S_{BET} m^2/g	S_{micro} m^2/g	V_{micro} m^3/g	D_{micro} nm
Ru_1/NC-700	0.8	286	-	-	-
Ru_1/NC-800	1.3	267	-	-	-
Ru_1/NC-900	1.6	216	23.2	0.01	0.8
Ru_1/NC-900-800NH_3	2.3	471	103.1	0.05	0.8

*The metal loadings were determined by ICP-OES.

Supplementary Table 2. The surface contents of various N species determined by XPS for the series of Ru_1/NC-T samples.

Entry	Catalysts	Atomic ratio of different N species (%)				
		Pyridinic N (%)	N-Ru moiety (%)	Pyrrolic N (%)	Graphitic N (%)	Oxidized N (%)
1	Ru_1/NC-700	52.6	17.4	22.4	6.5	1.1
2	Ru_1/NC-800	44.3	16.5	27.5	8.7	3.0
3	Ru_1/NC-900	33.4	15.1	25.6	22.1	3.8
4	Ru_1/NC-900-800NH_3	31.8	13.2	37.1	12.4	5.5

Supplementary Table 3. The superior performance of Ru_1/NC-900-800NH_3 as compared to the reported Ru and Rh nanocatalysts.[a]

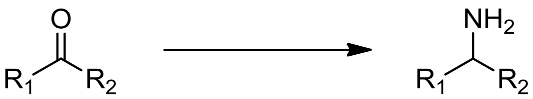

Entry	Catalyst	Molar ratio Ru/Substrate	P_{NH3} (MPa)	P_{H2} (MPa)	Time (h)	Tem. (°C)	Amine yield (%)	Production Rate ($g_{FAM} \cdot g_{Ru}^{-1} \cdot h^{-1}$)/ TOF (h^{-1})	Ref.
1	Ru_1/NC-900-800NH_3	1:400	0.5	2	4	100	97	170.7 / 176	This work
2[b]	Ru/Nb_2O_5	1:400	0.5	2	6	100	91	115 / 119	This work
3[c]	Ru/HZSM-5	1:400	0.5	2	7.5	100	68	89.6 / 92	This work
4[d]	Ru_1/NC/Nb_2O_5	1:400	0.5	2	3	100	97	227.6 / 235	This work
5	Ru/Nb_2O_5	1:250	0.1	4	2	90	99	124.2	1
6	Ru nanoparticles	1:250	8 mmol	2	1.5	90	99	125.2	2
7	Ru/TiP	1:500	0.3	1.7	24	30	91	18.2	3
8	Ru/HZSM-5	1:27	7 mmol	3	0.25	100	76	103.5	4
9	Ru/Nb_2O_5-L	1:1000	10 mmol	2	8	90	60	72.8	5
10	Ru/$Nb_2O_5 \cdot nH_2O$-300	1:250	8 mmol	4	4	70	89	54.6	6
11	Ru/SiO_2	1:670	1mL NH_4OH	7.5	4	130	90	145.5	7
12	Ru/γ-Al_2O_3	1:266	0.4	3	2	80	75	96	8
13	Ru/ZrO_2	1:40	29 mmol	2	12	85	94	1.9	9
14[e]	Ru/C	1:114	17.6mmol	1.5	14	100	95	13.8	10
15[f]	Ru/C	1:100	0.4	0.9	2	25	87.8	42.2	11
16[g]	Rh/Al_2O_3	1:2000	70mmol	2	2	80	91.5	45.8	12
17	$RuCl_2(PPh_3)_3$	1:50	0.5-0.7	4	24	130	85	2 / 2.1	13
18[h]	Ru/amino acid/diphosphine	1:400	0.5-0.7	5	18	130	94	22.2 / 20.9	14
19[i]	$Ru(PPh_3)_3H(CO)Cl$ + (S,S)-f-binaphane	1:100	2.5 equiv. NH_4I	3	24	80	84	4.4 / 3.5	15
20[j]	Ru[(s)-BINAP]$(OAc)_2$	1:100	5 equiv. NH_4OAc	5	24	80	94	7.7 / 3.9	16
21[k]	Ru(Cl)H(CO)$(PPh_3)_3$	1:100	0.6	4	16	120	99	7.4 / 6.2	17
22[k]	Trans-$[Ru(NH_3)_2\{PPh_2(2-OC_6H_4)\}_2]$	1:100	NH_4OAc	$NaBH_4$	48	90	100	2.5 / 2.1	18
23[k]	Ru(Cl)H(CO)$(PPh_3)_3$	1:100	0.6	4	16	120	99	7.4 / 6.2	19

[a] Unless otherwise noted, the substrate was furfural. [b] The catalyst was prepared based on the procedure reported in *J. Am. Chem. Soc.* 2017, **139**, 11493-11499; [c] The catalyst was prepared based on the procedure reported in *Mol. Cat.* 2020, **482**, 110755; [d] The catalyst was prepared with the similar procedure to that for Ru_1/NC-900-800NH_3 catalyst except for the employment of Nb_2O_5 support. [e] Glycolaldehyde as substrate; [f] (E)-1-(furan-2-yl)-5-methylhex-1-en-3-one as substrate; [g] Cyclohexanone as substrate; [h] Benzaldehyde as substrate; [i] Cyclohexyl methyl ketone as substrate; [j] Ortho-hydroxy-substituted diphenyl ketone as substrate; [k] Acetophenone as substrate; [l] Turnover frequency (TOF, h^{-1}) in parentheses.

Supplementary Table 4. Initial adsorption heats and saturated uptakes of H_2 on Ru_1/NC-T catalysts

Catalysts	H_2		H_2 after NH_3	
	Heat (kJ/mol)	Uptake (μmol/g)	Heat (kJ/mol)	Uptake (μmol/g)
Ru_1/NC-700	70.9	0.7	n.d.	n.d.
Ru_1/NC-800	91.3	3.7	45.5	2.0
Ru_1/NC-900	99.7	3.7	48.5	2.3
Ru_1/NC-900-800NH_3	115.8	7.9	67.3	4.1

Supplementary Table 5. Kinetic isotopic effect of H_2 and D_2 on reductive amination

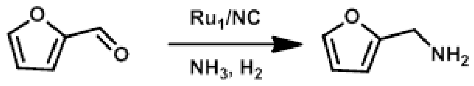

1a 2a

Entry	Catalysts	Yield of 2a (%)		KIE
		H_2	D_2	
1[a]	Ru_1/NC-900-800NH_3	18	16	1.1
2[b]	Ru_1/NC-900	21	12	1.7
3[b]	Ru_1/NC-800	19	9	2.1

[a]Reaction condition: 2 mmol furfural, 22 mg Ru_1/NC-900-800NH_3 (0.25mol% Ru, molar ratio: Ru:furfural=1:400), 0.5 MPa NH_3, 2 MPa H_2 or D_2, 100 ℃, 20 min, dodecane as internal standard; [b] 30min.

Supplementary Reference

[1] Komanoya, T., Kinemura, T., Kita, Y., Kamata, K. & Hara, M. Electronic effect of Ruthenium nanoparticles on efficient reductive amination of carbonyl compounds. *J. Am. Chem. Soc.* **139**, 11493-11499 (2017).

[2] Chandra, D. et al. A high performance catalyst of shape-specific ruthenium nanoparticles for production of primary amines by reductive amination of carbonyl compounds. *Chem. Sci.* **9**, 5949-5956 (2018).

[3] Xie, C. et al. Ambient-remperature synthesis of primary amines via reductive amination of carbonyl compounds. *ACS Catal.* **10**, 7763–7772 (2020).

[4] Dong, C. et al. Ru/HZSM-5 as an efficient and recyclable catalyst for reductive amination of furfural to furfurylamine. *Mol. Cat.* **482**, 110755 (2020).

[5] Guo, W., Tong, T., Liu, X., Guo, Y. & Wang, Y. Morphology-tuned activity of Ru/Nb_2O_5 catalysts for ketone reductive amination. *ChemCatChem*. **11**, 4130-4138 (2019).

[6] Deng, D., Kita, Y., Kamata, K. & Hara, M. Low-temperature reductive amination of carbonyl compounds over Ru deposited on Nb_2O_5 $\cdot$nH$_2$O. *ACS Sustainable Chem. Eng.* **7**, 4692-4698 (2019).

[7] S.Gould, N. et al. Selectivity control in catalytic reductive amination of furfural to furfurylamine on supported catalysts. *ChemCatChem.* **12**, 2106-2115 (2020).

[8] Dong, B. et al. Heterogeneous Ru-based catalysts for one-pot synthesis of primary amines from aldehydes and ammonia. *Catalysts.* **5**, 2258-2270 (2015).

[9] Liang, G. et al. Production of primary amines by reductive amination of biomass derived aldehydes/ketones. *Angew. Chem. Int. Ed.* **56**, 3050-3054 (2017).

[10] Jiang, S. et al. Direct Catalytic Conversion of furfural to furan-derived amines in the presence of Ru-based catalyst. *ChemSusChem.* **13**, 1699-1704 (2020).

[11] Song, S., Wang, Y. & Yan, N. A remarkable solvent effect on reductive amination of ketones. *Mol. Cat.* **454**, 87-93 (2018).

[12] Chatterjee, M., Ishizaka, T. & Kawanami, H. Reductive amination of furfural to furfurylamine using aqueous ammonia solution and molecular hydrogen: an environmentally

friendly approach. Green Chem. **18**, 487-496 (2016).

[13] Senthamarai, T. et al. Simple ruthenium-catalyzed reductive amination enables the synthesis of a broad range of primary amines. *Nat. Commun.* **9**, 4123 (2018).

[14] Pan, J., Zhang, R., Ma, S., Han, L. & Xu, B. Easily synthesized Ru catalyst efficiently converts carbonyl compounds and ammonia into primary amines. *ChemistrySelect.* **5**, 10933-10938 (2020).

[15] Ghosh, T., Ernst, M., K. Hashmi, A. S. & Schaub, T. Ruthenium catalyzed direct asymmetric reductive amination of simple aliphatic ketones using ammonium iodide and hydrogen. *Eur. J. Org. Chem.***2020**, 4796–4800 (2020).

[16] Hu, L., Zhang, Y. Zhang, Q., Yin, Q. & Zhang, X. Ruthenium-catalyzed direct asymmetric reductive amination of diaryl and sterically hindered ketones with ammonium salts and H_2. *Angew. Chem. Int. Ed.* **59,** 5321 –5325 (2020).

[17] Gallardo-Donaire, J. et al. Direct asymmetric Ruthenium-catalyzed reductiveamination of alkyl−aryl ketones with ammonia and hydrogen. *J. Am. Chem. Soc.* **140**, 355−361 (2018).

[18] Malan, F. P., Noh, J., Naganagowda, G., Singleton, Eric. & Meijboom, R. One-pot reductive amination of carbonyl compounds with ammonia via 'hydrogen borrowing' using hydrido- and bis-ammine P,O(Me)-ruthenacycles. *J. Organomet. Chem.* 825, 139-145 (2016).

[19] Gallardo-Donaire, J., Ernst, M., Trapp, O. & Schauba, T. Direct synthesis of primary amines via Ruthenium-catalysed amination of ketones with ammonia and hydrogen. *Adv. Synth. Catal.* 358, 358 – 363 (2016).
